# PGC-1*α* Mediated Peripheral Nerve Protection of Tongxinluo in STZ-Induced Diabetic Rats

**DOI:** 10.1155/2016/1287909

**Published:** 2016-07-18

**Authors:** Xiaopei Cui, Hua Feng, Xia Xu, Haijun Li, Hongyu Zhang

**Affiliations:** ^1^Department of Geriatrics, Qilu Hospital of Shandong University, 107 Wenhuaxi Road, Jinan 250012, China; ^2^Digestive Disease Department, Shandong Provincial Hospital, Shandong University, Jinan, Shandong 250021, China

## Abstract

*Aim*. To investigate the effect of Tongxinluo (Txl), a Chinese herbal compound, on diabetic peripheral neuropathy (DPN).* Methods and Results*. Diabetic rat model was established by peritoneal injection of streptozotocin (STZ). Txl ultrafine powder treatment for 16 weeks from the baseline significantly reversed the impairment of motor nerve conductive velocity (MNCV), mechanical hyperalgesia, and nerve structure. We further proved that Tongxinluo upregulates PGC-1*α* and its downstream factors including COX IV and SOD, which were involved in mitochondrial biogenesis.* Conclusion.* Our study indicates that the protective effect of Txl in diabetic neuropathy may be attributed to the induction of PGC-1*α* and its downstream targets. This finding may further illustrate the pleiotropic effect of the medicine.

## 1. Introduction

Diabetes mellitus is the most common cause of neuropathy in developed countries. Over 60% of diabetic patients suffer from diabetic peripheral neuropathy (DPN), leading to incapacitating pain, sensory loss, foot ulceration, poor wound healing, gangrene, and amputation [1].

DPN is characterized by nerve degeneration, especially in long axons of neurons. The pathologic changes of peripheral nerves include endoneurial microangiopathy, Schwann cell abnormality, axonal demyelination, and loss of fibers, resulting in reduction of motor and sensory nerve conduction velocity, as we and other groups previously reported [2, 3].

The Diabetes Control and Complications Trial (DCCT) already demonstrated that even optimal control of blood glucose could not prevent complications, suggesting that alternative treatment strategies are needed [4]. Increased production of free radicals or impaired antioxidant defenses is now a widely accepted participant in the development and progression of diabetic complications, including DPN [5]. The main source of reactive oxygen species (ROS) is the impaired respiratory chain of the mitochondria [6].

We also demonstrate abnormalities of mitochondrial structure in Schwann cells of sciatic nerves from STZ-induced diabetic rat models [2]. Mitochondrial malfunction correlates with the downregulation of mitochondrial proteins involved in respiratory chain in lumbar DRG in diabetes including cytochrome c oxidase subunit IV (COX IV, a complex IV protein) and NADH dehydrogenase Fe-S protein 3 (NDUFS3, a complex I protein) [6]. The upper stream transcriptional regulators related to mitochondrial biogenesis also decrease in diabetic muscle and kidney, for example, nuclear respiratory factor 1 (NRF-1) and peroxisome proliferator-activated receptor *γ* coactivator 1*α* (PGC-1*α*) [7, 8]. However, whether these regulators changed in diabetic neuropathy is still unclear.

Tongxinluo (Txl) is a traditional Chinese herb compound, comprising* Panax ginseng* and* Paeonia lactiflora *Pallas and so forth. It is approved by the State Food and Drug Administration of China for angina pectoris and ischemic stroke treatment for improving endothelial function and microvascular circulation [9, 10]. The possible mechanism includes suppression of oxidative stress [11] and activation of Akt-eNOS pathway [12]. Accumulating evidence showed multiple beneficial effect of Txl for diabetic complications. Txl ameliorates diabetic nephropathy by inhibiting miR-21 induced epithelial-to-mesenchymal transition and decreasing plasma endothelin concentration and local nephrin expression [13–15].

Newly published data also proved that Txl increases neuron growth factors including IGF-1, NGF, and BFGF and helps with nerve regeneration of diabetic rats [16]. It also inhibits cell apoptosis and suppresses p38MAPK phosphorylation of sciatic nerves in spontaneous type II diabetic KK/Upj-Ay mice [17]. But till now, the effect of Txl on mitochondrial function is uncertain.

In the present study, by STZ-induced diabetic rats model, we compare the mechanical allodynia, electrophysiology, morphology, PGC-1*α* expression, and mitochondrial biogenesis among control, diabetes, and 500 mg/kg/d Txl treated diabetic rats, to investigate the role of PGC-1*α* related mitochondrial biogenesis in the development of DPN and the effect of Txl on mitochondrial function.

## 2. Animal Model

30 male Wistar rats weighing 180–200 g (provided by Center for New Drugs Evaluation of Shandong University) were fasted overnight and diabetes mellitus was induced by a single injection of STZ (obtained from Sigma-Aldrich Corp., St. Louis, MO, USA) at a dose of 60 mg/kg peritoneally in a citrate buffer (pH 4.5). Age-matched rats in the control group (control, *n* = 10) received injection of normal sodium only. Tail vein blood glucose levels were measured 72 h after injection and the onset of the diabetic condition was defined as glucose level over 300 mg/dL. The diabetic rats were randomly divided into 2 groups: DM group, no treatment, *n* = 15, and Txl group, each rat receiving Txl ultrafine powder 500 mg/kg/d, *n* = 15. The preparation of Txl ultrafine powder (Shijiazhuang Yiling Pharmaceutical Company, China) has been described previously [18]. Txl contains 12 medicinal components:* Panax ginseng* C.A. Mey, 1.677%,* Ziziphus jujuba *Mill. var.* spinosa*, 1.173%,* Paeonia lactiflora* Pall., 1.558%,* Santalum album *L., 0.354%,* Dalbergia odorifera* T. Chen, 4.005%,* Boswellia carteri *Birdw., 5.927%, Borneolum Syntheticum, 3.626%,* Scolopendra subspinipes mutilans *L. Koch, 3.623%,* Buthus martensii *Karsch, 18.111%,* Steleophage plancyi*, 18.111%,* Hirudo nipponica*, 27.330%, and* Cryptotympana pustulata *Fabricius, 18.111%. Txl was given intragastricly at 9 am every day. All rats were fed a standard synthetic laboratory diet (provided by Center for New Drugs Evaluation of Shandong University) with ad libitum access to water for 16 weeks.

### 2.1. Estimation of Plasma Glucose Level

Blood samples were collected from the tip of the tails and plasma glucose level was estimated using standard assay kit (GOD-PAP, Sigma) once a week.

### 2.2. Measurement of Mechanical Allodynia

Mechanical algesia was determined by quantifying the withdrawal threshold of the hind paw in response to mechanical stimulation using von Frey hairs (BME-403, China; 1.19 g, 3.8 g, 5.8 g, 7.6 g, 10.12 g, 17.3 g, 52.0 g, and 73.0 g). The rat was placed in a hanging cage with a metal mesh floor and acclimated for at least 10 min. von Frey hair was manually applied to the plantar surface of the hind paw with increasing pressure until the filament bending to 90°; each test lasted less than 4 seconds. The test started from the 1.19 g von Frey hair and the one at which a paw withdrawal occurred was recorded. For each filament, the procedure was repeated 10 times and the pressure necessary to elicit 50% brisk foot withdrawal in response to this mechanical stimulus was interpreted as mechanical allodynia.

### 2.3. Electrophysiology

Nerve conductive velocity and amplitude of action potential were detected using BL-310 biomechanical system (BL-310, Taimen Co., Ltd., China). Animals were anesthetized with 10% Chloral Hydrate i.p. The left sciatic/tibia nerve was dissected rapidly and near nerve temperature was maintained at 37°C using liquid paraffin wax. The left sciatic/tibia nerve was placed in an insulated box, stimulated at the proximal end, and the action potentials were recorded at the distal end. The nerve was stimulated by square wave pulses (duration 0.1 ms, intensity 2 v). The average of 10 action potential traces was measured and the nerve length between stimulating electrode and recording electrode was recorded:

(1) MNCV  m/s=Distance  between  stimulating  and  recording  electrodeTibia  M  latency−Sciatic  M  latency.

### 2.4. Morphology

For microscope observation, the proximal portion of the sciatic nerve was biopsied at a constant site and immediately fixed in 4% paraformaldehyde and then embedded in wax. Transverse sections prepared for light microscope were stained with hematoxylin-eosin for axon observation and longitudinal sections were treated using Loyez staining method to observe the myelin.

For electroscope observation, the sciatic nerve sections were immediately fixed in 3% glutaraldehyde. The sections were then fixed with OsO_4_, dehydrated in ethanol, and embedded in SPI PON812 (SPI supplies: Division of Structure Probe, Inc., USA). Ultrathin (100 nm, 50 nm) transverse sections were transferred to 200-mesh formvar-coated copper grids and contrasted with 2% uranyl acetate in 70% ethanol followed by 0.3% sodium citrate before observing using H800 transmission electron microscope (Hitachi, H800, Japan).

### 2.5. mRNA Quantification

RNA was isolated from sciatic nerve using an RNeasy lipid kit (Qiagen, West Sussex, UK) according to the directions. Total RNA (1 *μ*g) was used to synthesize complementary DNA (cDNA) using SuperScript II Reverse Transcriptase, random primers, and dNTP (all from Invitrogen, Paisley, UK). Gene expression was quantified in duplicate using 1 *μ*L cDNA template by quantitative PCR (qPCR) on a 7500 Real-Time PCR System (Applied Biosystems, Carlsbad, CA, USA) using the appropriate gene expression assay as per the manufacturer's recommendations. Relative differences in gene expression between groups were determined using the 2^−ΔΔCt^ method [19]. The amplification efficiencies of the gene of interest and the housekeeping gene were equivalent.

### 2.6. Statistical Analysis

Results are shown as mean ± SEM. Statistical analysis was performed using SPSS13.0 software. Significance of difference between the groups was evaluated using Student's *t*-test. For multiple comparisons, one-way analysis of variance (ANOVA) was used. In case ANOVA shows significant differences, post hoc analysis was performed with Tukey's test or Dunnett test, and *p* < 0.05 was considered statistically significant.

## 3. Result

### 3.1. Influence of Txl on Glucose Level and Body Weight

**Figure 1:**
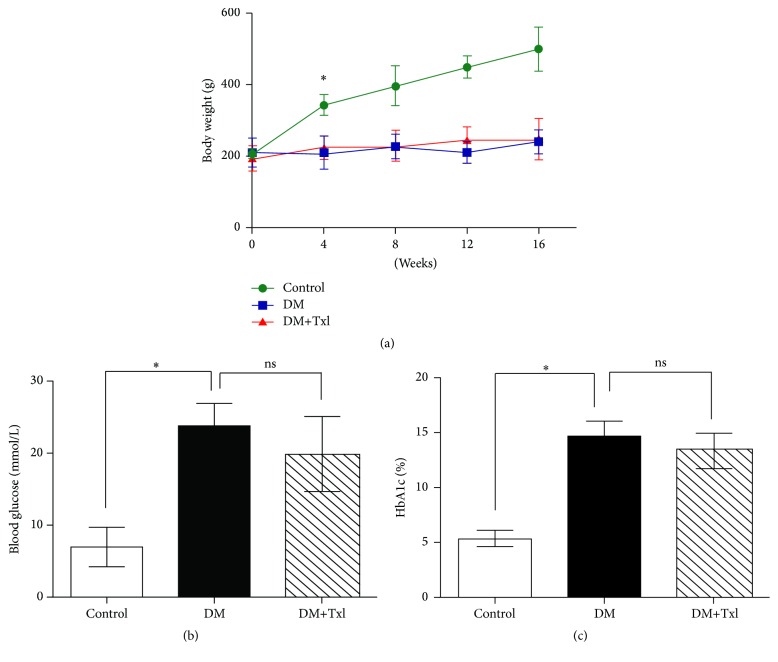
Body weight and blood glucose of the rats. (a) Body weight changes of the rats. The body weight of the control group increased gradually during the experiment. For diabetic groups, whether treated or not, the average body weight was much lower than control (^*∗*^ *p* < 0.01). (b) Free blood glucose comparison. The blood glucose was detected after 16 weeks at the end of the experiment. The DM group showed higher glucose level compared to normal control (^*∗*^ *p* < 0.01), whereas Tongxinluo did not affect blood glucose. (c) Glycosylated hemoglobin comparison. HbA1c was significantly high in the DM group than that in normal control (^*∗*^ *p* < 0.01), but there was no difference between Tongxinluo treated and untreated group.

The body weights and plasma glucose levels were similar at the baseline. 80% of the rats developed high glucose level 72 hours after STZ injection. The elevated plasma glucose in STZ-induced rats remained during the entire experiment; Txl did not affect blood glucose level when we detected both fasting glucose and glycosylated hemoglobin (HbA1c), as shown in Figures 1(b) and 1(c). Body weight of DM rats began to decrease since the 4th week, compared with normal control (*p* < 0.01), but Txl had no effect on it (Figure 1(a)).

### 3.2. Txl Improved MNCV in Diabetic Rats

**Figure 2:**
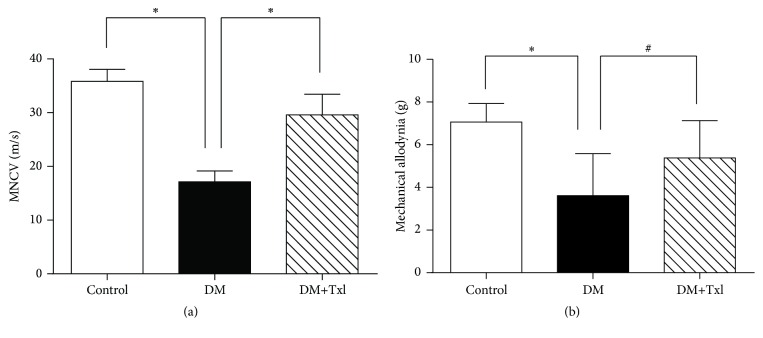
Nerve function estimation. (a) Effects of Tongxinluo on mechanical threshold of the rats. The diabetic rats in DM group showed apparent allodynia compared to control group (^*∗*^ *p* < 0.01), which was alleviated by Tongxinluo treatment in DM+Txl group (^*∗*^ *p* < 0.01). (b) Effects of Tongxinluo on motor nerve conductive velocity (MNCV) of the rats. The MNCV decreased in diabetic rats in DM group compared to control group (^*∗*^ *p* < 0.01), which was much improved by Tongxinluo treatment in DM+Txl group (^*∗*^ *p* < 0.01, ^#^ *p* < 0.05).

Diabetes caused significant reduction of sciatic-tibial MNCV compared with normal controls (17.13 ± 0.61 versus 35.83 ± 0.70 m/s, *p* < 0.01), while Txl treatment apparently prevented this slowing (29.59 ± 1.10 m/s, *p* < 0.01), as displayed in Figure 2(a).

### 3.3. Txl Alleviated Mechanical Allodynia in Diabetic Rats

We detected mechanical allodynia by measuring the hind paw withdraw threshold in response to von Frey hair. Diabetic rats exhibited apparent mechanical allodynia; the pressure required to elicit paw withdrawal was much lower than normal control (3.62 ± 0.59 g versus 7.06 ± 0.28 g, *p* < 0.01), while the allodynia was ameliorated with Txl treatment, and the pressure required to elicit paw withdrawal was 5.38 ± 0.5 g, *p* < 0.05, as shown in Figure 2(b).

### 3.4. Effects of Txl on Nerve Morphology

**Figure 3:**
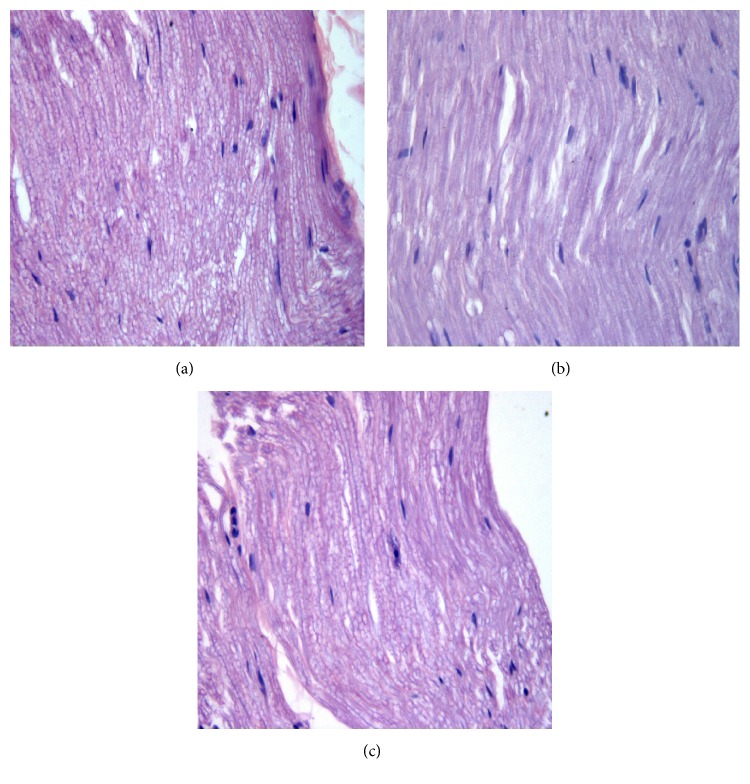
Light micrographs of the sciatic nerves, HE staining, ×400. Compared to control group (a), there was apparent segmental demyelination of nerves from diabetic rats in DM group (b) under microscope, which was reversed by Tongxinluo treatment in DM+Txl group (c).

**Figure 4:**
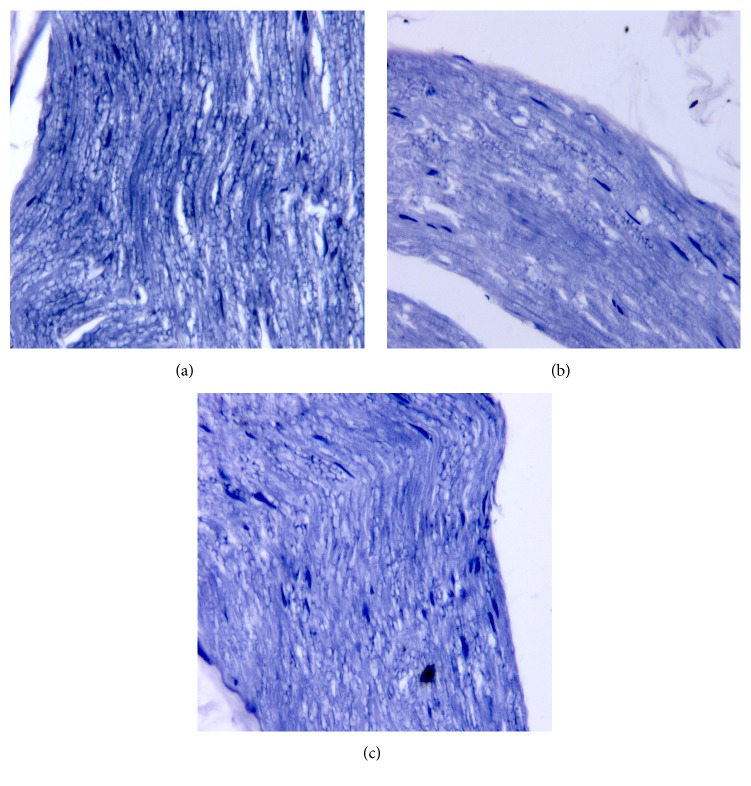
Light micrographs of the sciatic nerves, Loyez staining, ×400. Compared to control group (a), there was apparent segmental demyelination of nerves from diabetic rats in DM group (b) under microscope, which was reversed by Tongxinluo treatment in DM+Txl group (c).

There was apparent segmental demyelination of nerves from diabetic rats using both HE staining (Figure 3(b)) and Loyez staining (Figure 4(b)) under microscope. This demyelination was alleviated in Txl treated group as we have seen in Figures 3(c) and 4(c).

**Figure 5:**
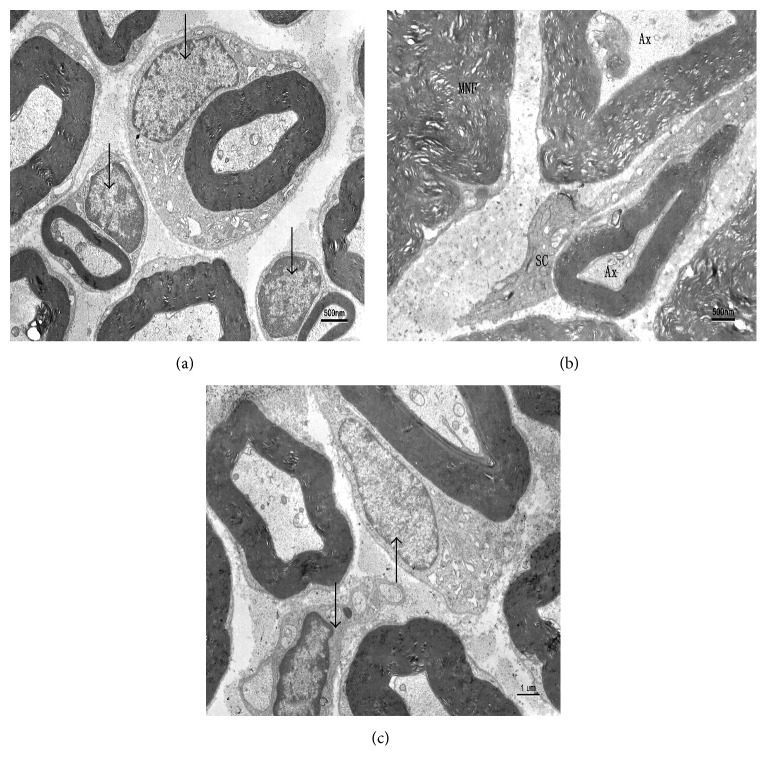
Electron micrographs of myelinated fibers of sciatic nerves. Compared to control group (a), the nerves in diabetic rats of DM group (b) showed depressed axons by thickened tunica vaginalis, crenulate Schwann cells with higher electron density, much fewer organelles, impaired basement membranes, and irregular nuclei. The ultrastructures were almost normal by Tongxinluo treatment in DM+Txl group (c). Arrows indicate Schwann cells.

Under electron microscope, normal nerves showed compact and integrated myelin arranged in concentric cycles with regular appearance and well-distributed electron density, well-arranged microfilaments, and microtubules and normal mitochondria arranged in the axons. Schwann cells surrounding the axons had well-distributed nuclear chromatin, organelles, and integrated basement membrane (Figure 5(a)). The nerves from untreated diabetes showed obvious derangement of the myelin with disconnected layers and much lighter electron density. Axons were depressed by thickened tunica vaginalis with more glia and much less microfilaments, microtubules, and mitochondria. The proliferated Schwann cells became granulated and exhibited higher electron density, fewer organelles, impaired basement membrane, and irregular nuclei with chromatin massively aggregated to the edge. With Txl treatment, the structures of Schwann cells and myelin were almost normal and, more importantly, the content and structure of mitochondria were preserved (Figure 5(c)).

### 3.5. Effects of Txl on Mitochondrial Biogenesis

**Figure 6:**
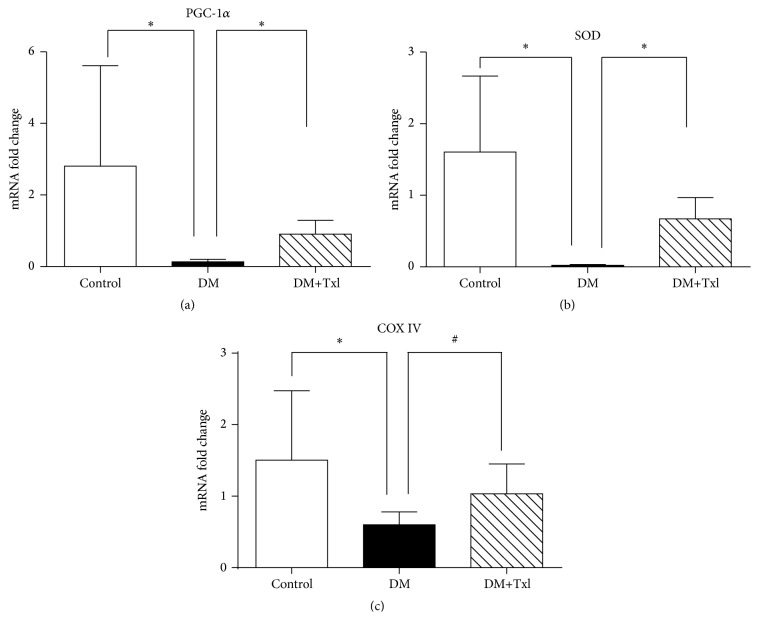
mRNA expression of genes associated with mitochondrial biogenesis of sciatic nerves. The mRNA level of PGC-1*α* (a), SOD (b), and COX IV (c) of sciatic nerves from all three groups was analyzed by RT-PCR. The expression of these genes in diabetic rats significantly decreased compared to control group (^*∗*^ *p* < 0.01), which was reversed by Tongxinluo treatment in DM+Txl group (^*∗*^ *p* < 0.01, ^#^ *p* < 0.05).

**Figure 7:**
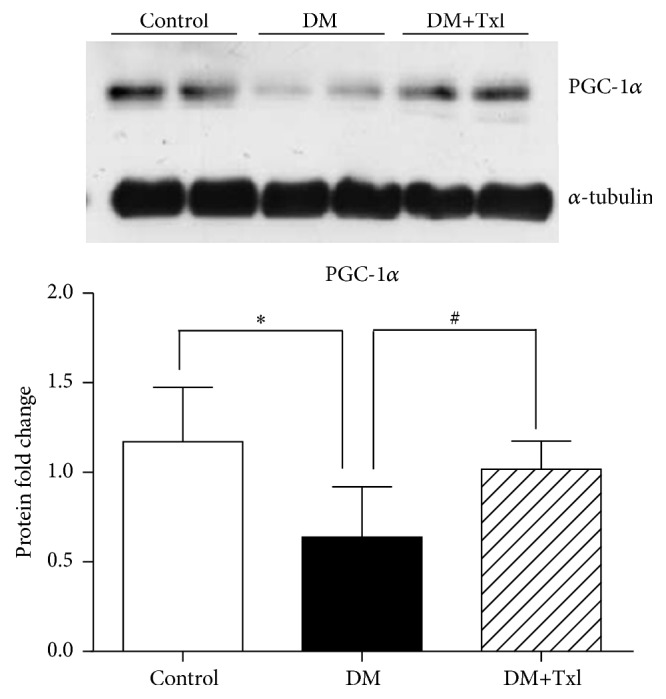
PGC-1*α* protein level of sciatic nerves. The protein level of PGC-1*α* was measured by western blot. The DM group showed decreased PGC-1*α* expression compared to normal control (^*∗*^ *p* < 0.01), whereas Tongxinluo induced PGC-1*α* expression (^#^ *p* < 0.05).

The mRNA level of molecules associated with mitochondrial biogenesis was detected by Real-Time PCR, including COX IV, SOD, and the upstream regulator PGC-1*α*. Both COX IV and SOD mRNA levels decreased in diabetic group compared to normal controls (*p* < 0.01), which were reversed by Txl treatment (*p* < 0.01) as shown in Figure 6. Meanwhile, both the mRNA and protein levels of PGC-1*α* showed the same trend (Figure 7).

## 4. Discussion

Diabetic neuropathy is the most common cause of peripheral neuropathies. Diabetic neuropathy is characterized with a reduction of motor and sensory nerve conduction velocity and a wide range of structural changes in peripheral nerves, including abnormal Schwann cell pathology, axonal degeneration, paranodal demyelination, and loss of myelinated and unmyelinated fibers, as we noticed in this study, consistent with the other groups. The region most affected by diabetic neuropathy is located in Schwann cell rich sciatic nerves, where striking upregulation of mitochondrial oxidative phosphorylation occurs [20].

Mitochondria play a key role in cell metabolism and also in free radical production and degradation. It is reckoned that high mitochondrial copy number (or higher mitochondrial mass) is protective for cells. Impaired mitochondria are the main source of reactive oxygen species (ROS) [21]. Mitochondrial dysfunction has been implicated in the pathophysiology of diabetic complications including muscle, heart, kidney, and nerves. In this study, we also observed mitochondrial abnormalities in peripheral nerves.

The upper stream transcriptional regulator related to mitochondrial biogenesis is PGC-1*α*, which increases COX IV and cytochrome* c* protein levels as well as the steady-state level of mtDNA [22]. PGC-1*α* coordinately regulates gluconeogenesis, glycolysis, lipogenesis, peroxisomal and mitochondrial fatty acid oxidation, and mitochondrial respiration efficiency. However, the change of PGC-1*α* in diabetic peripheral nerves is unclear. In this study, we found a decrease of PGC-1*α* expression in sciatic nerves of diabetic rats, which may be related to the downregulation of COX IV and SOD expression and malfunction of mitochondrial biogenesis. This malfunction was reversed by Txl treatment.

Tongxinluo (Txl) is a traditional Chinese herb compound, which consists of plant and insect products following a strict proportion for each portion. Pharmaceutical analysis has demonstrated that ginsenoside Rg1, ginsenoside Rb1, paeoniflorin, jujuboside A, and jujuboside B are main active components in Txl [18]. Txl is first proved by the State Food and Drug Administration of China for angina pectoris and ischemic stroke treatment and also has been demonstrated as beneficial for diabetic complications due to its multiple biologic effects [13, 23].

Txl has been proven to provide protective effects on peripheral nerves due to its induction of neuron growth factors and inhibition of nerve apoptosis through the MAPK pathway [17], which is associated with increased accumulation of ROS [24]. Here we also proved that Txl increases PGC 1*α* expression and therefore participates in mitochondrial biogenesis and energy metabolism; this finding may further illustrate the pleiotropic effect of the medicine.

## 5. Conclusion

In conclusion, Txl can apparently improve the decreased mechanical allodynia and sciatic-tibial nerve conductive velocity and alleviate nerve impairment of diabetic rats without affecting body weights and plasma glucose levels. The protective effect of Txl in diabetic neuropathy may be attributed to its induction of PGC 1*α* mediated mitochondrial biogenesis.
